# Effect of Precipitated Extracellular Marennine on Angiogenesis and Tumour Cell Proliferation

**DOI:** 10.3390/md23090364

**Published:** 2025-09-19

**Authors:** Mostefa Fodil, Javier Muñoz-Garcia, Amel-Khitem Benali, Jasmina Rogozarski, Virginie Mignon, Honora Labrana, Anna Lokajczyk, Pamela Pasetto, Jean-Luc Mouget, Catherine Boisson-Vidal, Dominique Heymann

**Affiliations:** 1 Laboratoire Biologie des Organismes, Stress, Santé, Environnement (BiOSSE), Faculté des Sciences et Techniques, Le Mans Université, Avenue Olivier Messiaen, F-72085 Le Mans, France; 2 Nantes Université, Centre National de la recherche Scientifique CNRS, UMR 6286, US2B, F-44322 Nantes, France; 3 Institut de Cancérologie de l’Ouest, Tumour Heterogeneity and Precision Medicine Lab., F-44805 Saint-Herblain, France; 4 Université Paris Cité, Therapeutic Optimization in Neuropharmacology, INSERM 1144, F-75006 Paris, France; 5 Université Paris Cité, Cellular and Molecular Imaging Facility, US25 INSERM, UMS3612 CNRS, F-75006 Paris, France; 6 Institut des Molécules et Matériaux du Mans (IMMM), UMR CNRS 6283, Le Mans Université, Avenue Olivier Messiaen, F-72085 Le Mans, France; 7 School of Medicine and Population Health, University of Sheffield, Sheffield S10 2RX, UK

**Keywords:** marennine, *Haslea ostrearia*, endothelial colony-forming cells, angiogenesis inhibitors, anticancer activity

## Abstract

Angiogenesis is a fundamental biological process involved in the formation of new blood vessels from the pre-existing vascular network. In addition to physiological processes, angiogenesis is also implicated in pathological conditions such as tumour growth and metastatic progression. Research on marennine, a water-soluble blue-green pigment produced by the marine diatom *Haslea ostrearia*, has highlighted various promising biological activities. In vivo studies have suggested the potential of marennine in cancer treatment. However, these studies were conducted with crude extracts, the exact composition of which remained poorly defined. In this context, our study aimed to explore the effects of marennine on angiogenesis and tumour proliferation by using a *Precipitated Extracellular Marennine* (*PEMn*) extract. Our results confirmed the antiproliferative properties of PEMn on several cancer cell lines associated with angiogenic tumours. We then analysed its impact on the key steps of the angiogenic process, including Endothelial Colony-Forming Cells (ECFCs) proliferation, migration, and tubulogenesis. In parallel, we investigated the underlying mechanisms of its action, notably by assessing its effects on cell cycle regulation, senescence, and apoptosis. PEMn significantly inhibited tumour cell proliferation, induced ECFC senescence and apoptosis, impaired migration and tubulogenesis, and downregulated VEGFR-1 expression, highlighting its potential as a novel marine-derived antiangiogenic compound. These findings provide deeper insights into the mechanisms of action of marennine, identifying this bioactive natural compound as a novel bioactive compound in cancer treatment.

## 1. Introduction

Microalgae play a crucial role in aquatic ecosystems by contributing to primary productivity and generating over 50% of atmospheric oxygen [1]. They also form the basis of marine food webs and participate in numerous ecosystem services. Due to their remarkable metabolic flexibility and ability to adapt to diverse environmental conditions, they have garnered increasing interest for their biotechnological and medical applications. Numerous studies have highlighted the production of bioactive metabolites with antimicrobial, antioxidant, antiviral, and antitumour properties [1,2,3,4]. These natural molecules hold significant potential for the development of novel therapeutic agents.

Among the microalgae of interest, the marine diatom *Haslea ostrearia* stands out due to its production of a water-soluble blue-green pigment, marennine, which is responsible for the greening of oyster gills in the Marennes-Oléron region [5,6]. This characteristic has made it the subject of scientific investigation for several decades, although the exact chemical structure of marennine remains to be fully elucidated. Determining its structure is particularly challenging due to its high molar mass, estimated at 9.8 kDa by mass spectrometry and confirmed by two-dimensional NMR [7]. Early hypotheses suggested similarities with anthocyanins or chromoproteins, but more recent studies have revealed a more complex structure, consisting of an exopolysaccharide linked to a chromophore, the exact nature of which remains unknown [6,8,9]. These studies have demonstrated that a fraction of marennine is composed of 1,3-glucan, a natural polysaccharide with remarkable pharmacological properties. Indeed, due to their triple-helical structure, 1,3-glucans play a key role in immune modulation and exhibit notable pharmacological effects, including antitumour, antibacterial, and wound-healing properties [10,11]. The similarity between marennine and these polysaccharides could explain some of its previously reported biological activities [12,13,14].

Research on marennine has highlighted various promising biological activities. Aqueous extracts of this molecule have demonstrated antioxidant, antibacterial, antiviral, and antiproliferative effects [7,12,13,14]. More specifically, these extracts have been shown to inhibit the proliferation of several human tumour cell lines, including SKOV-3 (ovarian cancer), SW116 (colon cancer), and M113 (melanoma) [12,13,14]. Moreover, in vivo studies have reported an inhibitory effect on the growth of pulmonary xenografts, suggesting an interesting therapeutic potential in cancer treatment. However, these studies were conducted with crude extracts, the exact composition of which remained poorly defined.

Angiogenesis is a fundamental biological process involved in the formation of new blood vessels from the pre-existing vascular network. This phenomenon is tightly regulated by complex interactions among endothelial cells, angiogenic growth factors, and the extracellular matrix [15,16]. It plays a key role in various physiological processes but is also implicated in pathologies such as tumour growth and metastatic progression. Among the cells involved in angiogenesis, endothelial colony-forming cells (ECFCs) stand out for their strong vasculogenic capacity and their role in the neovascularisation of ischaemic or tumour tissues [17]. Previous studies have relied on crude extracts with undefined composition, which limited mechanistic insights. In this study, we evaluate purified PEMn for the first time, obtained through a standardised precipitation method, allowing us to precisely assess its antiangiogenic and antitumour effects. In this context, our study aimed at exploring the effects of marennine on angiogenesis and tumour proliferation. In contrast to previous studies, we tested a purified extract of *Precipitated Extracellular Marennine* (PEMn), obtained using a recently developed protocol [18,19]. Initially, we confirmed the antiproliferative properties of PEMn on several cancer cell lines associated with angiogenic tumours. The tumour cell lines selected (melanoma, lung, glioblastoma, prostate, colon, and breast) are representative of angiogenesis-dependent cancers frequently used in preclinical antiangiogenic research. We then analysed its impact on the key steps of the angiogenic process, including ECFCs proliferation, migration, and tubulogenesis. In parallel, we investigated the underlying mechanisms of its action, notably by assessing its effects on cell cycle regulation, senescence and apoptosis. These findings will provide deeper insight into the mode of action of marennine and may pave the way for the development of new therapeutic strategies exploiting bioactive natural compounds in cancer treatment.

## 2. Results

### 2.1. Effect of PEMn on Tumour Cell Adhesion and Proliferation

**Figure 1:**
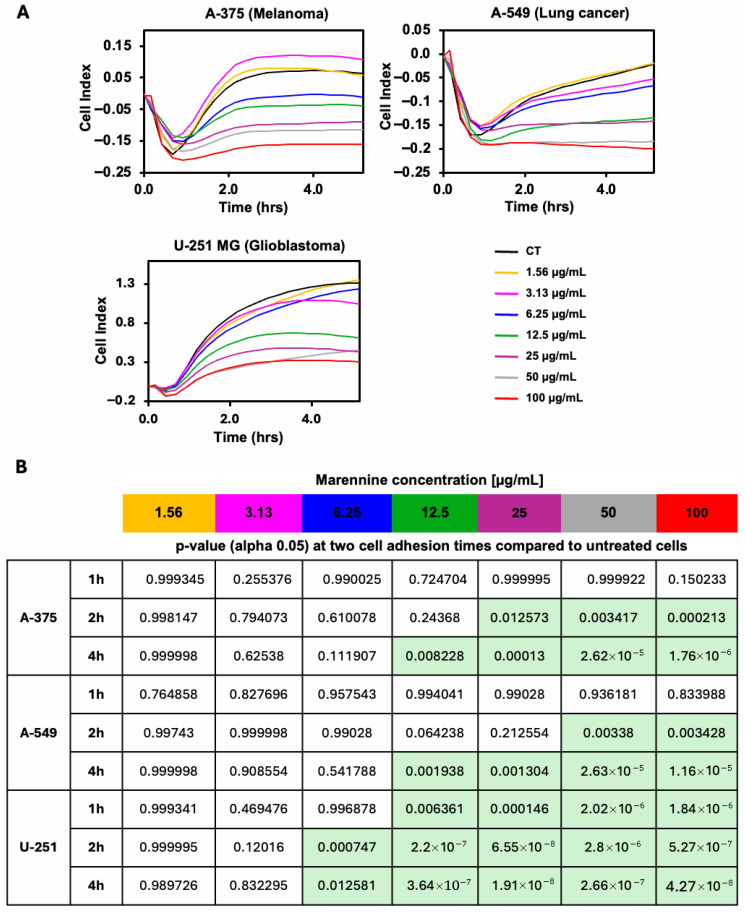
Cell adhesion of melanoma, lung cancer, and glioblastoma tumour cells is impaired in the presence of PEMn. (**A**) Human melanoma (A-375), lung cancer (A-549), and glioblastoma (U-251 Mg) cell lines were seeded for 5 h in the presence of increasing doses of PEMn (1.56, 3.13, 6.25, 12.5, 25, 50, and 100 µg/mL). Cell adhesion was followed during 5 h by real-time measurement of cell impedance using xCELLigence technology (RTCA Instruments). Lines represent the mean ± SD values of triplicate data. (**B**) *p*-values (alpha 0.05) of data (**A**) at three different times of cell adhesion (1 h, 2 h, and 4 h) compared to untreated cells (black line) curves. Light green boxes represent significant cell proliferation reduction/inhibition with respect to control cells. Statistical data was performed by use of ANOVA Tukey’s HSD test. Data are presented as mean ± SD (*n* = 3).

The potential antitumoural activity of the PEMn (*Precipitated Extracellular Marennine*) was assessed in several angiogenic tumour cell lines using xCELLigence technology, following an approach similar to that described in [19]. We first focused on the impact of PEMn on tumour cell adhesion (Figure 1). Three different angiogenic tumour cell lines, A-375 (human melanoma), A-549 (lung cancer) and U-251 (glioblastoma) were exposed to increasing concentrations of PEMn (ranging from 1.56 to 100 µg/mL), and cell adhesion was monitored over a 5 h period. As shown in Figure 1A, adhesion of the three tumour cell lines was impaired in a dose-dependent manner compared to untreated control cells (CT, black line). Cell adhesion was analysed in detail at three different time points: (i) global cell population adhesion, (ii) early adhesion (1 h post-seeding), (iii) mid-adhesion (2 h), and (iv) full cell population adhesion (4 h) (Figure 1B). The A-375 melanoma and A549 lung cancer cell lines showed a similar pattern of sensitivity, with disruption of adhesion observed at 1 h, regardless of the PEMn concentration. After 2 h post-treatment, higher doses of PEMn (50 and 100 µg/mL) significantly inhibited cell adhesion (*p* < 0.01 vs. control). After 4 h of treatment, 50% of the cells failed to adhere at 12.5 µg/mL of PEMn (*p* > 0.01), and less than half of the cell population adhered at higher doses (*p* < 0.001). The U-251 glioblastoma cell line showed a higher sensitivity to PEMn than the previous cell lines, with significant inhibition of cell adhesion observed from 12.5 µg/mL upwards as early as 1 h after treatment (*p* < 0.01 vs. control). These results suggest that PEMn can inhibit or slow down tumour cell adhesion in several angiogenic tumour types.

**Figure 2:**
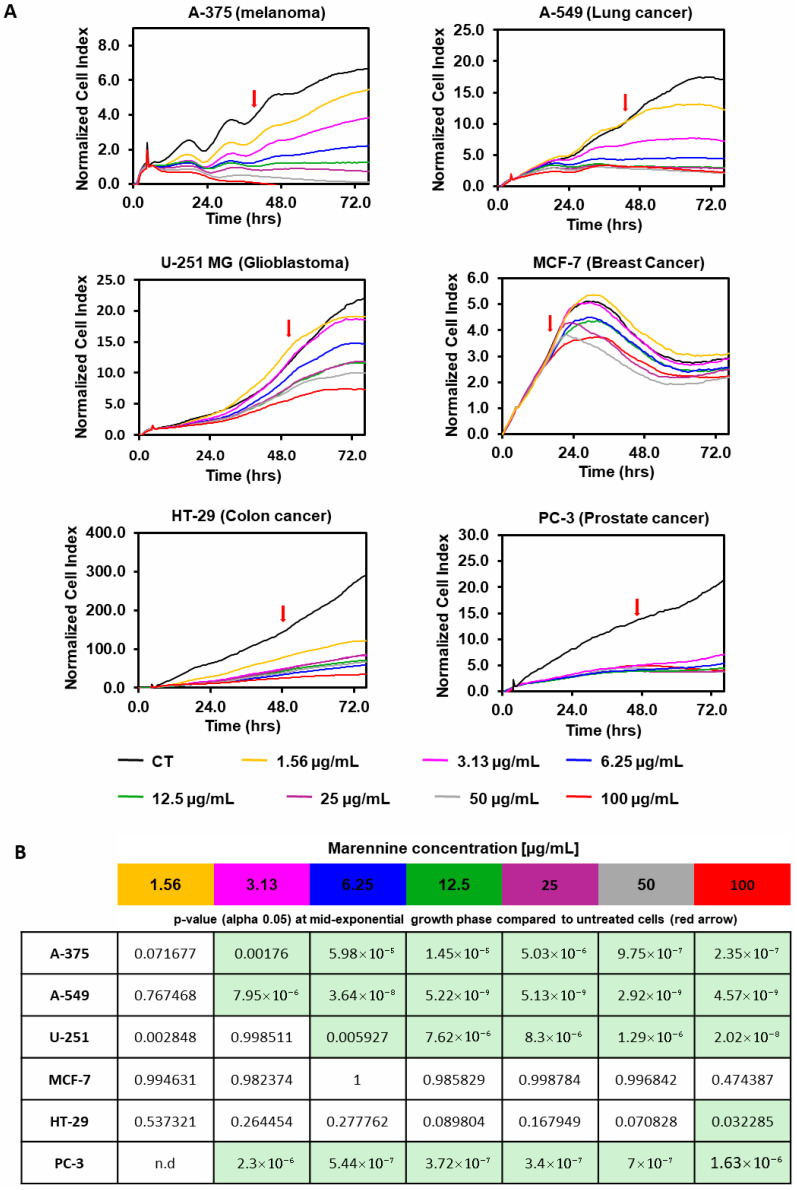
PEMn induces a reduction or arrest of tumour cell proliferation in a dose-dependent manner. (**A**) Human melanoma (A-375), lung cancer (A-549), glioblastoma (U-251 MG), breast cancer (MCF-7), colon cancer (HT-29), and prostate cancer (PC-3) cell lines were seeded for 4 h prior to the addition of increasing doses of PEMn (1.56, 3.13, 6.25, 12.5, 25, 50 and 100 µg/mL). Cell proliferation was followed during 72 h by real-time measurement of cell impedance using xCELLigence technology (RTCA Instruments). Proliferation curves were normalised to the time of drug incorporation. Lines represent the mean value of triplicate data. The red arrow represents the mid-exponential growth time in control, untreated cells. (**B**) *p*-values (alpha 0.05) of data from (**A**) compared to mid-exponential growth time (red arrow in A) of untreated cells (black line) curves. Light green boxes represent significant cell proliferation reduction/inhibition with respect to control cells. Statistical data was performed by use of ANOVA Tukey’s HSD test. Data are presented as mean ± SD (*n* = 3).

Similarly, the effect of PEMn in cell proliferation of several angiogenic tumour cell lines was assessed using xCELLigence technology. After 4 h of cell adhesion, six different tumour cell lines (melanoma A-375, lung cancer A-549, glioblastoma U-251 MG, breast cancer MCF-7, colon cancer HT-29 and prostate cancer PC-3A) were exposed to increasing doses of PEMn (from 1.56 to 100 µg/mL) and cell proliferation was monitored over 72 h (Figure 2). Except for the breast cancer cell line MCF-7, PEMn treatment resulted in reduced cell proliferation at most tested concentrations (Figure 2A). After 72 h, PEMn concentrations above 25 µg/mL resulted in an inhibition of cell proliferation of more than 90% in the three tested cell lines A-375, A-549 and PC-3. Compared to the mid-point of the exponential proliferation curves of the corresponding untreated group (CT, black line), where cells were in their optimal proliferation phase, PEMn significantly inhibited or slowed cell proliferation at the minimal dose of 3.13 µg/mL in A-375, A-549, and PC-3 cell lines (*p* < 0.01 vs. control) (Figure 2B). In the case of the U-251 glioblastoma cell line, a significant reduction in proliferation was observed from 6.25 µg/mL of PEMn (*p* < 0.01 vs. control), while for the HT-29 colorectal line, only the highest PEMn concentration (100 µg/mL) induced a significant reduction in proliferation (*p* < 0.05 vs. control). Altogether, these results indicate that PEMn induces an inhibition or slowdown of tumour cell proliferation in various angiogenic tumours.

### 2.2. Effect of PEMn on ECFCs Viability and Senescence

**Figure 3:**
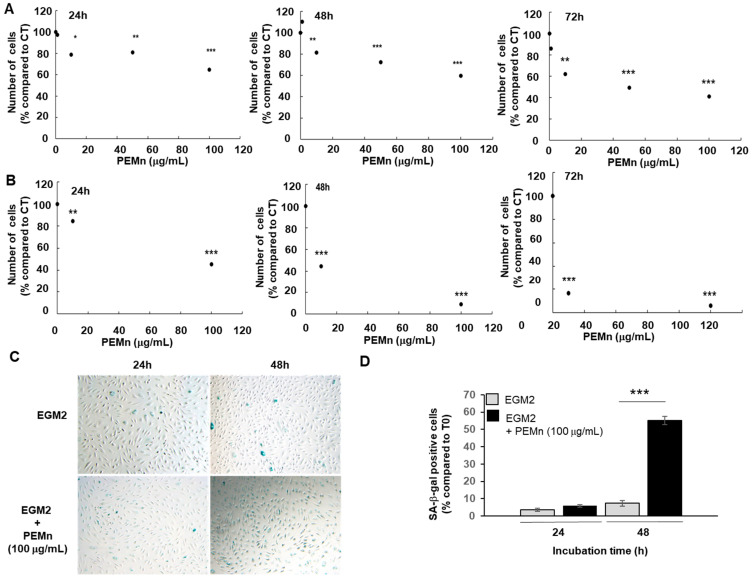
PEMn impairs ECFCs’ Proliferation and promotes senescence. ECFCs were cultured in EBM2 5% FBS culture medium supplemented with VEGF at 40 ng/mL in the presence or absence of different concentrations of PEMn (1, 10, 50 and 100 µg/mL), then counted ● after 24, 48, and 72 h of incubation. (**A**) This effect is dose-dependent. (**B**) PEMn reduces the viability of ECFCs. Cell numbers are normalised to the mean number of cells grown in the absence of PEMn (control condition, CT). (**C**,**D**) Cells were cultured in EGM2 (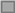) either alone or in the presence of 100 µg/mL of PEMn (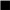, 100 µg/mL) for 24 and 48 h. Senescence was then assessed by senescence-associated-galactosidase (SA-β-gal) staining. SA-β-gal positive cells appear blue. Representative images are shown from one out of three independent experiments (phase contrast micrograph, original 10). (**D**) Cellular senescence was quantified as the number of SA-CTRL β-gal positive cells. Data are expressed as the mean ± SD from 3 independent experiments by use of one-way ANOVA and Student’s *t*-test analysis * *p* < 0.05, ** *p* < 0.01, and *** *p* < 0.001 vs. initial number of ECFCs at T0.

We then evaluated the effect of PEMn on the proliferation of ECFCs cultured under different conditions: an EBM2-5% FBS medium supplemented with VEGF (40 ng/mL), used as a control, and the same medium supplemented with different concentrations of marennine (1, 10, 50 and 100 µg/mL). The addition of VEGF was justified by the key role it plays in angiogenesis, in the microenvironment of the tumour, where it is present at high levels. As shown in Figure 3A, the addition of PEMn to the ECFCs culture medium significantly reduced their proliferation after 24 to 72 h of treatment. A dose-dependent inhibition was observed, starting at 10 µg/mL (20%) after 24 h and increasing to 100 µg/mL (34%). After 72 h, proliferation inhibition reached approximately 60% in the presence of 100 µg/mL PEMn. A decrease in cell viability was observed with increasing concentrations of PEMn, with an IC50 of 6.2 µg/mL at 72 h (Figure 3B). The lowest effective concentration (10 µg/mL) reduced cell viability to 16.4 ± 2.7% (*p* < 0.001 vs. vehicle) after 72 h of treatment. The maximum effect was observed at 100 µM PEMn, where viability dropped to 5.9 ± 0.6% (*p* < 0.001 vs. vehicle).

PEMn also induced morphological changes in ECFCs, including cell detachment and debris accumulation, visible from 24 h and worsening up to 72 h at 100 µg/mL (Figure S1, representative images showing dose- and time-dependent morphological alterations). A senescence-associated β-galactosidase (SA-β-gal) assay confirmed that PEMn treatment significantly increased senescence in ECFCs (Figure 3C,D). The percentage of senescent ECFCs was 2 times higher than in control cells after 24 and 72 h.

**Figure 4:**
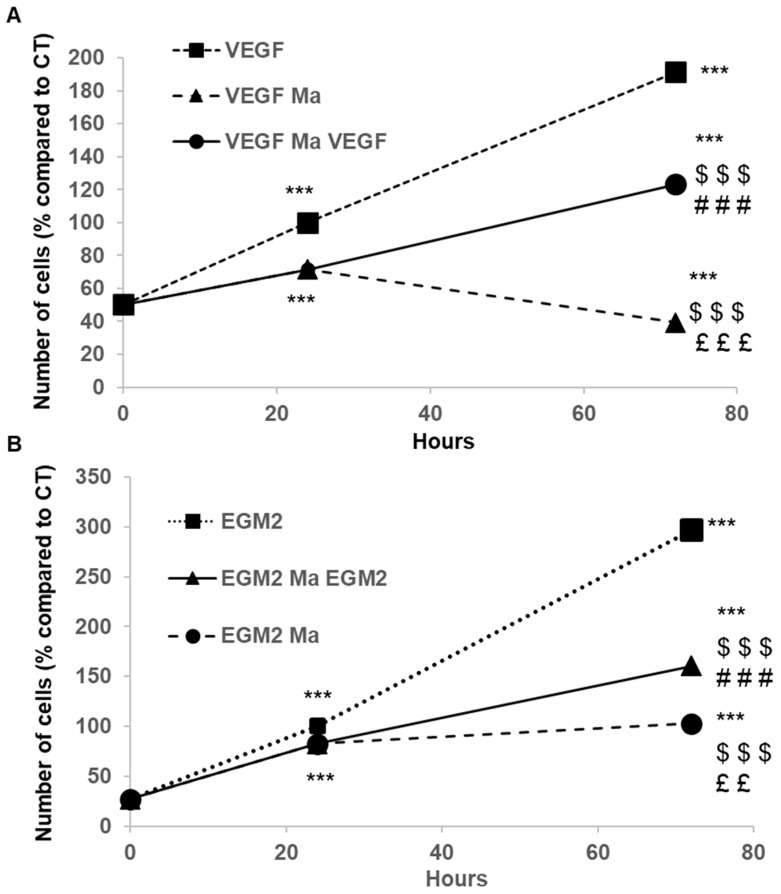
PEMn reduces ECFCs’ viability in a reversible manner. Cells were cultured in (**A**) EBM2 5% FBS VEGF (40 ng/mL (VEGF) or (**B**) EGM2 10% FBS (EGM2), either alone or in the presence of 100 µg/mL of PEMn (VEGF Ma or EGM2 Ma). After 24 h of treatment, the culture media were replaced, and a subset of cells was incubated for an additional 48 h in the control medium (EBM2 5% FBS VEGF (VEGF Ma VEGF) or EGM2 10% FBS (EGM2 Ma EGM2)) without PEMn. The number of cells was quantified using a pNPP colourimetric assay after 24 and 72 h of incubation. Results are expressed as a percentage of the negative control (% vs. CT) as the mean ± SD from 3 independent experiments by use of one-way ANOVA and Student’s *t*-test analysis *** *p* < 0.001 vs. control T0, $$$ *p* < 0.001 vs. VEGF or EGM2 at the same time of incubation, ### *p* < 0.001 vs. VEGF Ma or EGM2 Ma T24, ££ *p* < 0.01 and £££ *p* < 0.001 vs. VEGF Ma VEGF or EGM2 Ma EGM2 T72.

The inhibitory effect of PEMn is reversible (cytostatic). When PEMn is removed from the EBM2-5% FBS VEGF culture medium after 24 h of incubation, ECFCs’ proliferation resumes, reaching a level three times higher than under continuous exposure (Figure 4A). A similar effect is observed in EGM2 medium, although to a lesser extent Figure 4B. This difference is explained by the presence of numerous growth factors and cytokines, such as FGF, VEGF, and SDF-1 (CXCL12), in this medium.

### 2.3. PEMn Induces ECFCs Growth Arrest and Apoptosis

The antiproliferative effect of PEMn on ECFCs observed in our study is associated with cell cycle arrest in the G0/G1 phase and the induction of apoptosis. To assess the effect of PEMn on the cell cycle and apoptosis of ECFCs, we performed a cellular analysis 24 and 72 h after treatment with PEMn (100 µg/mL). Treatment of ECFCs with 100 µg/mL of PEMn for 24 and 72 h led to a decrease in the number of cells in the G2/M phase (*p* = 0.0491 and *p* = 0.0176, respectively), accompanied by a significant increase in the proportion of cells in G0/G1 (*p* = 0.0016 and *p* = 0.0002, respectively) (Table 1).

PEMn also induces a significant increase in apoptotic cell death. The percentage of late apoptotic cells (Annexin V^+^/PI^+^) is significantly higher in ECFCs exposed to 100 µg/mL of PEMn for 24 h compared to control groups (Table 2 and Figure S2, Flow cytometry analysis after 24 h and 72 h treatment with 100 µg/mL PEMn).

After 24 h of PEMn exposure, the proportion of late apoptotic cells in treated cultures was more than twice that observed in control conditions (27.8% versus 12.1%, respectively, *p* = 0.0091), indicating a marked induction of apoptosis. This was accompanied by a significant reduction in the proportion of viable cells (44.1% versus 69.4%, respectively, *p* < 0.0001), suggesting a loss of membrane integrity and cell survival. The pro-apoptotic effect of PEMn became even more evident after 72 h of treatment, with a further increase in the number of necrotic or dead cells compared to controls. These observations support the hypothesis that PEMn acts as a strong inducer of cell death through apoptotic mechanisms.

### 2.4. Effects of PEMn on ECFCs Migration

**Figure 5:**
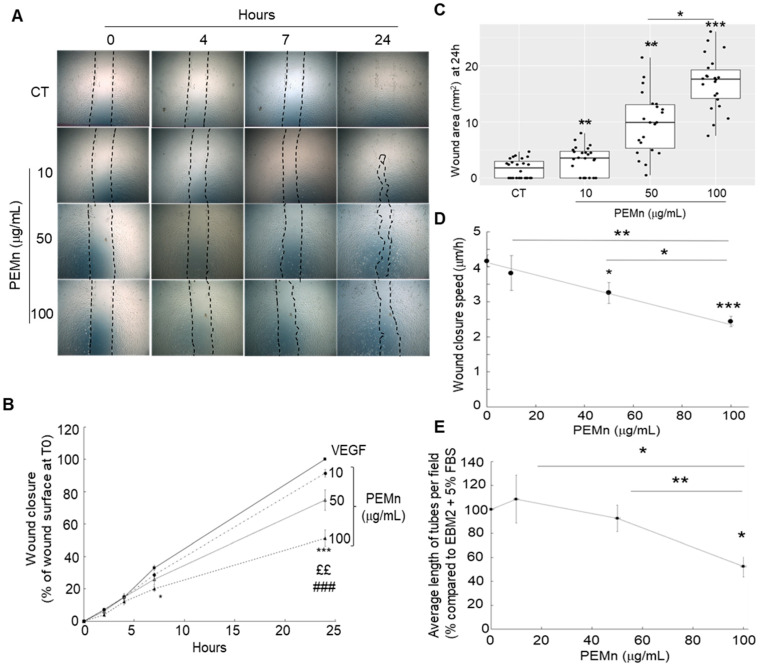
PEMn inhibits ECFCs’ migration and disrupts capillary-like structure formation. (**A**) ECFCs were cultured in EGM2 10% FBS until they reached confluence. Once confluence was achieved, cells were synchronised for 24 h, a wound was created, and the endothelial layer was incubated in EBM2 medium supplemented with 5% FBS (CT) and VEGF (40 ng/mL), in the presence or absence of PEMn (10, 50 and 100 µg/mL) for 2, 4, 7, and 24 h. Representative images: Light microscope images (10× magnification) of the endothelial layers were taken at T0 (immediately after wounding) and after 2, 4, 7, and 24 h of incubation for each condition: EGM2 (CT), EGM2 supplemented with increasing doses of PEMn. (**B**) Wound healing kinetics: The percentage of wound closure was calculated by normalising the wound area to that measured at T0 for each condition EBM2 5% FBS VEGF (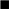) EBM2 5% FBS VEGF supplemented with PEMn 10 µg/mL (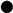) 50 µg/mL (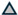) and 100 µg/mL (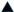). (**C**) Dose-dependent effect: After 24 h of incubation, the percentage of wound closure was calculated relative to T0 for each condition. (**D**) Average wound reduction speed (µm/h): Migration speed was determined from the wound reduction kinetics for each condition. The graphs represent the average migration speed from two replicates across four independent experiments. (**E**) PEMn reduces in vitro tubulogenesis. After synchronisation in EBM2-2% FBS, ECFCs were seeded onto Matrigel^®^ and incubated in EBM2-5% FBS VEGF (40 ng/mL), with or without PEMn (10, 50, 100 µg/mL). Representative images of vascular structure formation in vitro after 16 h of incubation in EGM2-5% VEGF (40 ng/mL), in the absence or presence of PEMn (10, 50, and 100 µg/mL), obtained using light microscopy (10× magnification). Dose–response curve of PEMn’s effect on the average tube length after 16 h of incubation, normalised to the control condition. Tube length was measured using the Histolab^®^ software (Microvision Instruments, Evry, France). Data are expressed as the mean ± SD from 5 independent experiments. 1 one-way or two-way ANOVA, Fisher correction * *p* < 0.05, ** *p* < 0.01 and *** *p* < 0.001 vs. VEGF-treated ECFCs, ££ *p* < 0.01 and ### *p* < 0.001 vs. VEGF-PEMn 10 µg/mL or VEGF-PEMn 50 µg/mL -treated ECFCs, respectively.

One of the key steps in angiogenesis is the migration of endothelial cells. To assess this process, we performed a wound healing assay. After creating a wound on a subconfluent cell layer, we monitored its closure over time. ECFCs were incubated in EBM2 medium supplemented with 5% FBS and VEGF (40 ng/mL) (control condition), with or without the addition of PEMn at 10, 50, and 100 µg/mL. To avoid experimental bias due to paracrine effects that could enhance migratory and/or proliferative capacities, only wells with an initial wound area ≥ 40 µm^2^ were considered. The results, presented in Figure 5, show that PEMn inhibits ECFCs migration in a time- (Figure 5A,B) and dose-dependent manner (Figure 5C). After 7 h of incubation, no significant difference is observed between the 10 and 50 µg/mL PEMn conditions and the untreated control (Figure 5A,B): approximately 30% of the wound is closed (32.9 ± 1.6% for the control, 28.6 ± 1.2% and 26.0 ± 3.3% in the presence of 10 and 50 µg/mL PEMn, respectively). However, at 100 µg/mL PEMn, wound healing is delayed, with a 40% reduction compared to the control (20 ± 1.6% *p* = 0.0117). After 24 h, the wound is completely closed in the control condition, whereas the inhibitory effect of PEMn is clearly visible (Figure 5A,B). This effect is observed from 50 µg/mL (74.9 ± 6.3% closure *p* = 0.0002) and is even more pronounced at 100 µg/mL (51.3 ± 5.1% *p* < 0.0001) compared to the untreated control (100% closure in EBM2 5% FBS VEGF).

PEMn treatment significantly slows wound reduction speed, resulting in a reduction of up to 48.7% compared to the control (Figure 5D). This speed is 3.2 ± 0.3 µm/h for 50 µg/mL PEMn, 2.4 ± 0.2 µm/h for 100 µg/mL, compared to 4.2 ± 0.2 µm/h for the control condition (EBM2 5% VEGF, 40 ng/mL). During the angiogenic process, endothelial cell differentiation is a key step leading to the formation of vascular structures. To assess the ability of ECFCs to form pseudotubes in vitro, they were seeded onto Matrigel^®^. Cells were incubated for 16 h in EBM2-5% FBS VEGF (40 ng/mL) medium, with or without PEMn (10, 50, 100 µg/mL). The inhibitory effect of PEMn was observed from 50 µg/mL and became particularly pronounced at 100 µg/mL, with an approximately 50% reduction in total tube length compared to the control condition (Figure 5E and Figure S3, Representative microscopic images showing reduced tube formation with increasing PEMn concentrations).

### 2.5. Effect of PEMn on ECFCs Cytokine Profiles

The levels of several proteins in cell lysates, including VEGF-R1, IL-1β, IL-6, and MMP-9, produced by ECFCs in response to PEMn treatment were quantified using the multiplex Luminex xMAP™ technology. Table 3 shows their expression levels after 24, 48, and 72 h of incubation. PEMn induced a time-dependent and statistically significant decrease in VEGF-R1 levels, with up to a 2.75-fold reduction observed after 48 h (*p* < 0.001) and a 1.75-fold reduction after 72 h (*p* = 0.0063). Conversely, PEMn treatment led to a marked increase in the inflammatory cytokines IL-6 (5.1-fold, *p* < 0.001) and IL-1β (29-fold, *p* < 0.001), as well as MMP-9 (30-fold, *p* < 0.001). These effects were significant at 72 h post-treatment.

## 3. Discussion

Natural products have always been an invaluable source of new bioactive compounds, particularly in the search for anticancer therapies. Among them, marine compounds stand out for their chemopreventive, chemotherapeutic and anti-angiogenic properties, playing a role in regulating cell proliferation, mitogenic signal transduction, and the formation of new blood vessels [20,21,22,23]. Despite considerable advances in oncology, treatment resistance, relapses, and side effects of conventional therapies remain major challenges. Therefore, new approaches are necessary, and nature, particularly microorganisms associated with plants and microalgae, represents a promising source of molecules with high therapeutic potential [24]. To date, nearly 60% of drugs used against cancer originate from natural sources [25]. In this context, marennine, a pigment produced by the microalga *Haslea ostrearia*, has garnered increasing interest for its antitumour activity. Studies have shown that aqueous extracts containing marennine exert cytotoxic activity on several cancer cell lines, particularly colorectal carcinoma cells and melanoma lines [12,14], as well as antitumour activity in vivo [8,13]. Its effect, which depends on concentration and cell type, leads to a decrease in cell viability after prolonged exposure. This anticancer potential could be explained by its action on oxidative stress and the induction of cell cycle arrest. Although its biological properties have been studied since the 1990s, its structure and mode of action remain to be clarified. Marennine exhibits structural and /or functional similarities with fucoidans and carotenoids, known for their anticancer effects, particularly on angiogenesis. In the present work, we evaluated the inhibitory effect of a purified marennine extract (PEMn) produced by *Haslea ostrearia* on several tumour cell lines. Our results confirm the cytotoxic activity of PEMn, in agreement with previous studies on aqueous extracts of marennine, resulting in an inhibition or reduction in tumour cell adhesion as well as cell proliferation. While adhesion assays confirmed PEMn inhibition of tumour cell adhesion, the molecular basis remains to be clarified. Future studies should address key adhesion molecules such as integrins (αvβ3, β1) and cadherins to further elucidate PEMn’s anti-adhesive properties.

**Figure 6:**
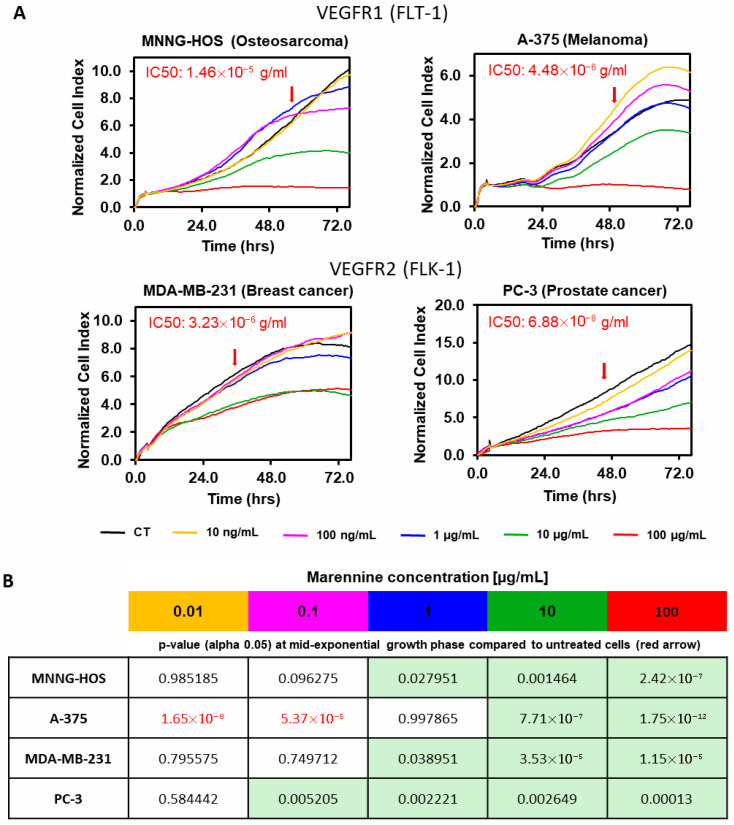
PEMn induces a reduction or arrest of tumour cell proliferation in a dose-dependent manner. (**A**) Human osteosarcoma (MNNG-HOS), melanoma (A-375), breast cancer (MDA-MB-231) and prostate cancer (PC-3) cell lines were seeded for 4 h prior to the addition of increasing doses of PEMn extract (0.01, 0.1, 1, 10, and 100 µg/mL). Cell proliferation was followed during 72 h by real-time measurement of cell impedance using xCELLigence technology (RTCA Instruments). Proliferation curves were normalised to the time of drug incorporation. IC50 was determined by the RTCA analysis software. Lines represent the mean value of triplicate data. The red arrow represents the mid-exponential growth time in control untreated cells. (**B**) *p*-values (alpha 0.05) of data from (**A**) compared to mid-exponential growth time (red arrow in **A**) of untreated cells (black line) curves. Light green boxes represent significant cell proliferation reduction/inhibition with respect to control cells. Statistical data was performed by use of ANOVA Tukey’s HSD test. Data are presented as mean ± SD (*n* = 3).

Angiogenesis is a key step in tumour and metastasis proliferation and dissemination [25]. VEGF and its receptors are highly expressed in patient tumour samples as well as in standard tumour cell lines [25]. We decided to determine the effect of PEMn and its IC50 during the proliferation of several cell lines characterised by the overexpression of VEGF receptors as Flt-1 (VEGFR-1) in osteosarcoma MNNG-HOS [25] and melanoma A-375 cell lines [25], and Flk-1 (VEGFR-2) in breast cancer MDA-MB-231 [25] and prostate cancer PC-3 cell lines [25]. As previously, cell proliferation of tumour cells was monitored by xCELLigence technology in the absence or presence of increased doses of PEMn (from 10 ng/mL to 100 µg/mL) for 72 h. In all cell lines tested, a significant reduction in cell proliferation was induced by PEMn (Figure 1, Figure 2, Figure 6 and Figure S4 proliferation curves from Figure 2 and Figure S5 proliferation curves from Figure 3). No major differences in reduction or inhibition of cell proliferation were observed between cell lines that overexpress Flt-1 (VEGF-R1) or Flk-1 (VEGF-R2) receptors. However, cell lines overexpressing VEGF-R1 receptors showed a lower IC50, 68.8 ng/mL for PC-3 and 3.23 µg/mL for MDA-MB-231 (*p* < 0.5 vs. control), compared to those that overexpress VEGF-R1, 14.6 µg/mL for MNNG-HOS and 4.48 µg/mL for A-375 (*p* < 0.5 vs. control). Since these cell lines induced angiogenic tumours, we then investigated the impact of PEMn on key angiogenesis-related processes in vitro, using circulating endothelial progenitor cells (ECFCs). Our results show that PEMn inhibits ECFCs proliferation in a dose-dependent manner, an effect potentially linked to interference with VEGF-mediated signalling. Indeed, this effect is observed when the cells are cultured in the presence of VEGF (40 ng/mL). However, it appears to be transient: once PEMn is removed from the culture medium, the inhibition is reversed, suggesting that marennine acts extracellularly—either by reducing VEGF bioavailability or by interacting with its receptor. Future studies will include receptor phosphorylation assays to confirm whether PEMn directly interferes with VEGFR-2 or VEGFR-1 signalling. These further tests will target the VEGFR-2/SRC/FAK and VEGFR-2/MEK/ERK signalling pathways, which are needed to elucidate the precise mechanism. Ligand-binding and phosphorylation assays will help identify whether PEMn interacts directly with VEGFR-1 or VEGFR-2 or indirectly modulates their downstream signalling. Also, it would be interesting in future validation studies to include known VEGFR inhibitors such as sunitinib as positive controls for direct comparison. Further studies will be needed to determine whether PEMn also affects VEGF secretion by tumour cells. To date, only a few microalgal compounds—mainly carotenoids and polysaccharides—have demonstrated inhibitory effects on endothelial cell proliferation during angiogenesis [26]. For example, astaxanthin, a carotenoid with strong antioxidant properties, exerts antiproliferative effects on the ECV304 cell line, widely used for modelling angiogenic behaviour [27]. More recently, fucoxanthin has been reported to inhibit proliferation in human lymphatic endothelial cells [28]. Compared to fucoxanthin, astaxanthin, and fucoidans, PEMn uniquely combines inhibition of angiogenesis with induction of ECFC senescence and VEGFR-1 downregulation.

In addition to its antiproliferative effect, PEMn significantly reduced ECFCs migration in wound healing assays and inhibited the formation of vascular-like structures on Matrigel^®^. Astaxanthin has also shown antimigratory effects, though primarily under hypoxic conditions. It would thus be relevant to explore PEMn activity under such conditions to better mimic the tumour microenvironment. These effects suggest a possible alteration of cytoskeletal dynamics. Supporting this, our Luminex multiplex analysis showed an increase in MMP-9 levels in the culture medium. Additional studies are required to explore the mechanistic links between cytoskeletal reorganisation and PEMn-induced inhibition of angiogenic behaviours. These antiangiogenic effects were associated with a significant reduction in ECFCs viability, increased cellular senescence, and cell cycle arrest in the G0/G1 phase, accompanied by a decrease in the S and G2/M phases. PEMn also induced a substantial increase in apoptosis, indicating activation of both cytostatic and cytotoxic mechanisms. Apoptosis is a desirable therapeutic endpoint in anticancer strategies, and this aligns with the known pro-apoptotic effects of marennine in tumour models [29]. Even if the binding of the marennine remains to be elucidated, marennine can be uptaken and internalised by ECFCs (Figure S6, Confocal microscopy showing progressive intracellular accumulation of PEMn (green fluorescence) over time).

PEMn treatment also led to a significant downregulation of VEGF-R1 expression, alongside a marked increase in proinflammatory cytokines (IL-6, IL-1β) and MMP-9. The reduced VEGF-R1 expression may reflect a functional reprogramming of ECFCs or a negative feedback loop aimed at desensitising the cells to proangiogenic stimuli. VEGF-R1 is known to act in part as a decoy receptor for VEGF-A, modulating its availability to VEGF-R2, which more directly drives proliferative and migratory signalling. Thus, downregulation of VEGF-R1 may contribute to angiogenesis modulation by diminishing this regulatory “brake.” Alternatively, it could indicate a loss of active endothelial identity consistent with PEMn-induced functional impairment. The marked increase in IL-6 and IL-1β levels suggests a cellular inflammatory response to PEMn-induced stress. These cytokines are commonly secreted during apoptosis, oxidative stress, or innate immune activation. Their upregulation may involve mitochondrial dysfunction, oxidative imbalance, or activation of inflammatory signalling cascades such as NF-κB. Furthermore, this profile is reminiscent of the senescence-associated secretory phenotype (SASP), which includes IL-6 and IL-1β as key effectors. Multiplex Luminex analysis also revealed a strong upregulation of MMP-9, a metalloproteinase involved in extracellular matrix degradation and tissue remodelling. MMP-9 is frequently elevated in contexts of inflammation, cellular stress, and tissue injury. Its expression is known to be induced by IL-1β and IL-6, suggesting a convergent regulatory network between cytokine signalling and matrix remodelling. In our model, the increased MMP-9 levels may contribute to the inhibition of vascular structure formation by promoting local matrix disorganisation. This inflammatory and degradative profile is consistent with the overall antiangiogenic effect of PEMn and supports the hypothesis of a stress-induced reprogramming of ECFCs. Although IL-6, IL-1β, and MMP-9 are typically pro-angiogenic mediators, PEMn simultaneously increased their levels while inhibiting angiogenesis. This apparent paradox may reflect a stress-induced senescence-associated secretory phenotype (SASP), in which cells secrete inflammatory mediators but exhibit impaired angiogenic functionality. Moreover, elevated MMP-9 may destabilise extracellular matrix integrity, preventing the formation of stable vascular structures. Investigating the involvement of SASP or other stress-induced transcriptional programmes would provide further insight into the nature of the cellular response to PEMn.

These findings are consistent with earlier observations showing that aqueous extracts of *Haslea ostrearia*-containing marennine induce G1/S cell cycle arrest in NSCLC-N6 lung cancer cells [13]. Similarly, carotenoids extracted from microalgae have been shown to induce cytostasis and apoptosis in osteosarcoma cells via caspase activation [30]. Together, these results support the notion that PEMn exerts its bioactivity through interconnected pathways involving inhibition of proliferation, migration, angiogenesis, and induction of apoptosis, senescence, and inflammatory signalling in endothelial progenitor cells.

In conclusion, the purified marennine extract exhibits strong potential as an anticancer molecule, particularly due to its effects on angiogenesis and cell proliferation. *Haslea ostrearia* could thus represent a promising source for the development of new therapeutic agents. However, further studies are essential to precisely characterise its structure, understand its mechanism of action, and evaluate its safety for potential clinical applications. Future studies will validate PEMn efficacy in vivo using mouse tumour and neovascularization models. It would also be interesting to address drug delivery systems such as nanoparticles to enhance PEMn bioavailability, alongside pharmacokinetics and pharmacodynamics studies to support clinical development.

## 4. Materials and Methods

### 4.1. Microalgae and Purification of Marennine

*Haslea ostrearia* diatoms used for biomass production and pigment extraction were derived from samples collected in Bourgneuf Bay, France. The blue pigment was extracted and purified as previously described [18]. To sum up, *Haslea ostrearia* strain Nantes Cultures Collection (NCC) 495 was cultured at 16 ± 1 °C, with an irradiance of µ100 mol.m^−2^ s^−1^ provided by Philips TLD 36 W/965 fluorescent tubes with an alternance cycle of 14 h light/10 h dark. Cultures were grown with autoclaved artificial seawater, prepared from a commercial sea salt mix (Instant Ocean, Aquarium Systems^®^, Mentor, OH, USA), pH 7.6 0.2, salinity 32 ppm, with an enrichment solution as described by Mouget et al. [31]. To remove cell debris, the culture medium was filtered through 15 µm and 1.4 µm cut-off paper filters. The filtered supernatant was then concentrated by a specific acid-base precipitation procedure described in French patent no. (FR2019/052933). The blue precipitate formed was gathered by centrifugation (4000 rpm, for 5 min) and dissolved with formic acid. This concentrated blue extract (*PEMn: Precipitated Extracellular Marennine*) was dialysed and then further purified using a 20 g C-18 solid phase extraction cartridge (Fischer Scientific, Illkirch, France). The marennine was recovered using a 1:1 water–ethanol mixture. The mixture was then evaporated in order to recover the purified extract of marennine as a dry powder, which was then solubilized with sterile water at a stock concentration of 1 mg/mL. PEMn preparations were tested for endotoxin contamination using the LAL Kinetic Chromogenic Assay (Lonza, Verviers, Belgium), with results below detection limits. Purified pigment was stored, protected from light, at 4 °C. This solution was used for the experiments planned for the project.

### 4.2. Cell Isolation and ECFCs Culture

Umbilical cord blood provided by AP-HP, Hôpital Saint-Louis, Unité de Thérapie Cellulaire, CRB-Banque de Sang de Cordon (Paris, France) was collected after normal full-term deliveries with the written informed consent of the mother. Mononuclear cells were isolated from human cord blood by density-gradient centrifugation on Pancoll. They differentiate into endothelial cells in angiogenic growth factor–rich medium, with initial colonies appearing between days 10 and 14 and the first passage around day 20. As previously shown, despite differences in outgrowth, ECFCs and mature endothelial cells, HUVECs differ in the expression of the hematopoietic stem cell marker CD133, which is rapidly down-regulated during ECFC differentiation and reaches HUVEC-like levels by day 60. Obtained ECFCs were identified by their characteristic morphology, then by immunostaining for von Willebrand factor, combined expression of endothelial markers (CD31, KDR, Tie-2, CD144), and double-positivity for DiI-AcLDL uptake and BS-1 lectin binding [32]. One day before the experiments, ECFCs were growth-arrested for 18 h in EBM2, 2% Foetal Calf Serum (FCS, starvation medium, Lonza, Brussels, Belgium) and released from growth arrest by adding EBM2, 5% FCS (basal medium), with or without 1–100 µg/mL of PEMn in the presence or absence of VEGF (40 ng/mL, Abcys, Paris, France) at 37 °C 5% CO_2_. Cells were used for assays between passages 4 and 6 (approximately 30–40 days of culture).

### 4.3. Tumour Cell Lines and Cell Culture

All human tumour cell lines used in the present study were obtained from the American Tissue Cell Collection (ATCC, Molsheim, France) and from the European Collection of Authenticated Cell Cultures (ECACC, Salisbury, UK) and were tested routinely as mycoplasma-free. All experiments were performed at 37 °C in a humidity-saturated controlled atmosphere and 5% CO_2_. A375 melanoma, MNNG-HOS osteosarcoma, and U-251 MG glioblastoma lines were cultured with DMEM 4.5 g/L high glucose with pyruvate (Gibco, ThermoFisher Scientific, Illkirch-Graffenstaden, France). LnCap prostate adenocarcinoma cell line was expanded in RPMI1640 (Gibco, ThermoFisher Scientific, France). A-549 non-small cell lung cancer and PC-3 prostate cancer cell lines were cultured with DMEM/F-12 (Gibco, ThermoFisher Scientific, France). MDA-MB-231 breast carcinoma cell line was cultured with Leibovitz’s L-15 media (Gibco, ThermoFisher Scientific, France). The HT-29 colorectal adenocarcinoma cell line was cultured in McCoy’s 5A medium (Gibco, ThermoFisher Scientific, France). MCF-7 breast adenocarcinoma cell line was cultured in Minimum essential medium supplemented with sodium pyruvate and NEAA (Gibco, ThermoFisher Scientific, France). All culture media were supplemented with 2 mM L-glutamine (Gibco, ThermoFisher Scientific, France) and 5% of FBS (Eurobio-scientific, Les Ulis, France).

### 4.4. Real-Time Cell Proliferation Assay

Cell proliferation was analysed by xCELLigence technology (Agilent, Les Ulis, France) as previously described [19]. Background was measured by adding 50 μL of corresponding media into an E-Plate view 96 (Agilent, Santa Clara, CA, USA). Before the beginning of treatment, cells were seeded in triplicate at 8000 to 10,000 cells (depending on the cell line) per well (50 μL) for 4 h at 37 °C before adding increasing concentrations of PEMn (from 100 μg/mL to 10 ng/mL). Concentration ranges (10 ng/mL–100 µg/mL) were selected based on preliminary IC_50_ determinations and previous studies with crude marennine extracts. Proliferation curves were normalised with respect to the time point of drug incorporation. The plate was monitored for 72 h after treatment using a RTCA instruments (Agilent) using a RTCA device (Agilent). Experiments were conducted in triplicate.

### 4.5. Real-Time Cell Adhesion Assay

Cell adhesion was analysed by xCELLigence technology (Agilent, Les Ulis, France) similarly as described for cell proliferation. Background was measured by adding 50 μL of corresponding media into an E-Plate view 96 (Agilent). Then, cells were seeded in triplicate at 8000 per well (50 μL) and in the presence or absence of PEMn (from 100 μg/mL to 1.56 μg /mL, 100 μL). Cell adhesion into the plate was monitored for 5 h at 37 °C using a RTCA instruments (Agilent) using a RTCA device (Agilent). Experiments were conducted in triplicate.

### 4.6. In Vitro Angiogenesis Assay and Viability

To investigate the effect of various concentrations of PEMn on ECFCs’ proliferation and tubular morphogenesis, ECFCs were stimulated as described above. Cell outgrowth and in vitro tube formation were evaluated as previously described [32]. Cell viability determined by measuring acid phosphatase activity (pNPP, Sigma) at 405 nM (Fluostar optima; BMG Labtech, Champagny S/Marne, France) [33] was calculated as a percentage of vehicle-treated control cells considered 100% viable. In preliminary experiments, no significant influence of solvents on cell proliferation and morphology was observed.

### 4.7. Senescence

ECFCs were incubated in EGM2 (control) or EGM2 + PEMn (EGM2 Ma, 100 μg/mL) for 24 h at 37 °C and fixed in the presence of a galactose derivative (X-Gal) hydrolysed by β galactosidase, an enzyme overexpressed in senescent cells. The oxidised indolic moiety of the hydrolysed X-Gal forms a blue precipitate. The wells were photographed, and the percentage of blue (senescent) cells was evaluated and compared according to the culture conditions.

### 4.8. Cell Cycle Analysis

Analysis was performed by FACS. Permeabilized ECFCs are treated with propidium iodide (PI), a DNA intercalator that can be used to determine cell cycle phase. Combined with annexin V, which detects phosphatidylserine exposed following loss of plasma membrane asymmetry during apoptosis, PI can be used to quantify necrotic, apoptotic, or healthy ECFCs. Briefly, ECFCs were treated with PEMn (100 µg/mL) for 24–72 h and then harvested, washed, and fixed in 100% ethanol on ice for 30 min at −20 °C. After centrifugation, the cell pellets were washed, resuspended in phosphate-buffered saline (PBS), and incubated with RNase to prevent PI from binding to RNA, and stained with PI at room temperature for 30 min in the dark. DNA content was analysed using a FACS flow cytometer (BD LSR Fortessa^TM^ Becton Dickinson, NJ, USA).

### 4.9. Cell Apoptosis Analysis

Apoptosis was detected with an annexin V-fluorescein isothiocyanate (FITC) kit (TACS^TM^ Annexin V-FITC Apoptosis detection Kit R&D Systems, Inc., Abingdon, UK) according to the manufacturer’s instructions. The cells were stimulated as described above (Section 4.2). Briefly, after serum deprivation, ECFCs were incubated for 24 to 72 h in the presence or absence of 100 µg/mL of PEMn, then collected, washed twice with ice-cold PBS, and resuspended in ice-cold binding buffer before addition of annexin V-FITC and propidium iodide (PI) solutions. The tube was incubated for 15 min at room temperature in the dark, before being analysed by flow-cytometry (BD LSR Fortessa^TM^ Becton Dickinson). The percentage of apoptotic cells was determined using FlowJo^TM^ Engine v4.00770 software (Beckton Dickinson). Cells stained Annexin V^+^/PI^−^ were considered in early apoptosis, while cells stained Annexin V^+^/PI^+^ were defined as in late apoptosis, indicating progression from early apoptosis with membrane permeabilization. Cells stained Annexin V^−^/PI^+^ were considered necrotic or dead.

### 4.10. Wound Healing Assay

Migration was evaluated by wound scratch assays. ECFCs were seeded in 6-well plates and incubated with serum. After 24 h, scratch wounds were created in the confluent monolayer using a sterile 200 µL pipette tip.  After removal of floating cells, cells were stimulated as described above (Section 4.2). Cell migration into the wound space was estimated at 0, 4, 7, and 24 h after wounding using an inverted microscope (Nikon, France) equipped with a digital camera and analysed using the NIH ImageJ software (NIH, Bethesda, MD, USA). Wound closure was determined as the difference between wound width at 0 and 24 h.

### 4.11. Cytokine and Growth Factor Multiplex Analysis

The experiments were conducted on both untreated control ECFCs and ECFCs treated with PEMn. The supernatant from the cell culture medium was collected in microtubes and immediately frozen at −80 °C for subsequent analysis. A bead-based antibody mix targeting IL-1β, IL-6, IL-8, VEGF-R1, VEGF-R2, and MMP-9 (Human Luminex^®^ Discovery Assay Bio-Techne SAS, Noyal-Chatillon-sur-Sèche, France) was added to a 96-well filter plate containing standards and culture medium from treated or control cells. Analyte levels were measured according to the manufacturer’s instructions. Fluorescence specific to R-PE-conjugated beads was quantified using a Bio-Plex^®^ 200 system (Bio-Rad, Minneapolis, MN, USA). Each condition was tested in triplicate.

### 4.12. Statistical Analysis

Experiments were analysed using Prism 6 (GraphPad Prism 6 software; La Jolla, CA, USA). Data are expressed as the mean ± SD of at least 5 independent experiments. One-way analysis of variance (ANOVA) and Student’s *t*-test were used to identify significant differences between the control and experimental groups. A probability (*p*) value of <0.05 was considered statistically significant. Independent experiments have been performed in triplicate, and data are given as a mean ± SD. Results were considered significant at *p* values ≤ 0.05. Corresponding groups were compared using ANOVA and Tukey’s HSD test. The data analysis for this paper was generated using the Real Statistics Resource Pack software (Release 9.4.5). Copyright (2013–2025) Charles Zaiontz. www.real-statistics.com (accessed on 25 September 2024).

## Figures and Tables

**Table 1:** Quantification of cell population distribution over the various stages of ECFCs’ cycle.

PEMn in ECFCs (µg/mL)	G0/G1 (%)	S (%)	G2/M (%)
24 h incubation			
0	35.0 ± 6.2	22.1 ± 2.4	28.7 ± 6.7
100	59.2 ± 5.0 ^b^	15.0 ± 3.1	14.3 ± 5.0 ^a^
72 h incubation			
0	46.4 ± 3.9	24.3 ± 4.3	20.0 ± 3.6
100	66.9 ± 2.1 ^c^	17.3 ± 2.8	7.8 ± 1.1 ^a^

ECFCs were incubated in either EGM2 10% FBS or EGM2 10% FBS containing PEMn (100 µg/mL) for 24 and 72 h before harvesting. After washing and fixation in 70% ethanol on ice, ECFCs were treated with RNAse and stained with propidium iodide (PI). DNA content was analysed using a FACS flow cytometer. Data are expressed as the mean ± SD from 5 independent experiments. one-way ANOVA and Student’s *t*-test analysis ^a^ *p* < 0.05, ^b^ *p* < 0.01, ^c^ *p* < 0.001 vs. vehicle-treated ECFCs.

**Table 2:** Statistical analysis of the percentages of early apoptotic (Annexin V-stained cells, early apop) and late apoptosis (Annexin V + IP-stained cells, late apop) cells was expressed as a percentage of the total cell number.

ECFCs	Q4 Live (%)	Q3 Early Apop (%)	Q2 Late Apop (%)	Q1 Dead (%)
24 h incubation				
Control	73.6 ± 3.3	12.1 ± 4.3	8.7 ± 1.0	5.7 ± 1.6
PEMn 100 µg/mL	53.4 ± 2.8 ^c^	15.2 ± 2.2	19.9 ± 2.3 ^b^	11.5 ± 2.6
72 h incubation				
Control	69.4 ± 5.1	10.3 ± 3.3	12.1 ± 2.6	8.4 ± 1.7
PEMn 100 µg/mL	44.1 ± 7.1 ^c^	10.2 ± 1.3	27.8 ± 6.0 ^b^	17.8 ± 2.0

Data are expressed as the mean ± SD from 5 independent experiments and analysed using one-way ANOVA and Student’s *t*-test. ^b^ *p* < 0.01, ^c^ *p* < 0.001 vs. vehicle-treated ECFCs.

**Table 3:** Effects of PEMn treatment (100 µg/mL) on VEGF-R1, IL-6 (C), IL-1β (D), and MMP-9 (E) levels in the ECFCs lysate after 24, 48, and 72 h of incubation.

	24 h		48 h			72 h		
Variables (pg/mL)	EGM2	PEMn	EGM2	PEMN	*p*-Value	EGM2	PEMn	*p*-Value
VEGF-R1	2.70 ± 0.26	1.31 ± 0.15	6.12 ± 0.06	2.20 ± 0.02	<0.001	6.04 ± 0.11	3.47 ± 0.48	<0.001
IL-6	47.00 ± 1.75	55.64 ± 4.53	86.68 ± 1.79	96.63 ± 2.34	0.8030	88.30 ± 1.04	453.04 ± 60.95	<0.001
IL-1β	0.04 ± 0.00	0.04 ± 0.00	0.05 ± 0.00	0.04 ± 0.00	0.9792	0.05 ± 0.00	1.47 ± 0.44	<0.001
MMP-9	0.17 ± 0.01	0.27 ± 0.1	0.27 ± 0.05	0.16 ± 0.01	0.9084	0.21 ± 0.01	6.3 ± 2.10	<0.001

Results are presented as mean ± standard deviation from triplicate measurements, *p* < 0.001, baseline vs. vehicle-treated ECFCs for each time on incubation. The values indicated in bold were statistically significant. one-way ANOVA and Student’s *t*-test.

## Data Availability

Data and materials are available from the corresponding author upon request.

## Supplementary Materials

The following supporting information can be downloaded at: https://www.mdpi.com/article/10.3390/md23090364/s1. Figure S1: PEMn induces morphological changes in ECFCs. ECFCs were cultured in EBM2 + 5% FBS culture medium supplemented with VEGF at 40 ng/mL in the presence or absence of increasing concentrations of PEMn (1, 10, 50, and 100 µg/mL). ECFCs were then observed under a microscope after 24, 48, and 72 h of incubation. Representative images are shown. Figure S2: PEMn increases apoptotic cell death in ECFC. Determination of apoptotic cell death using flow cytometry. Following 24 h and 72 h treatment with PEMn (100 µg/mL), ECFC were stained with PI (y-axis) and Annexin V (x-axis). The percentage of apoptotic cells was determined using a FACS flow cytometer. (A) Control group. (B) Experimental group (100 µg/mL PEMn). Q1: dead cells (IP-stained cells); Q2: late apoptotic cells (Annexin V + IP-stained cells); Q3: Live cells; and Q4: early apoptotic cells (Annexin V-stained cells). Figure S3: Representative microscopic images of the tube formation assay to illustrate the inhibitory effect of PEMn on in vitro tubulogenesis. ECFCs were cultured in EBM2 medium supplemented with 5% FBS and VEGF (40 ng/mL) in the absence (control) or presence of PEMn (10, 50, and 100 µg/mL) for 4 h and 24 h. In the control condition, a decrease in tube network length was observed after 24 h as a result of syncytia fusion (i.e., areas delineated by the tubes). Under both incubation times (4 h and 24 h), PEMn treatment markedly inhibited tubulogenesis, with a strong effect already evident at 50 µg/mL. White scale bar: 20 µm (10× magnification). Figure S4: Proliferation curves from Figure 2 with mean values ± S.D. *n* = 3. Figure S5: Proliferation curves from Figure 3 with mean values ± S.D., *n* = 3. Figure S6: Internalisation of PEMn by ECFCs. ECFCs were cultured in the presence of 500 mg/mL of mareninne for 30 min and 2 h. After incubation time, cells were fixed and stained for observation under a fluorescent microscope. DNA (nuclei) was stained by TO-PRO3 (dilution 1/500, Life Technologies, France, ref#T3605), and the actin network by using Phalloidin Alexa Fluor 555 (dilution 1/50, Invitrogen, France, ref#A34055). Stained cells were observed under a Leica TCSSP8 confocal microscope. Internalisation of mareninne (green fluorescence) is visible after 30 min of incubation time and increases progressively during time, as shown after 2 h of incubation. Original magnification: X63.

## Abbreviations

CI	Cell Index
ECFCs	Endothelial Colony-Forming Cells
FBS	Foetal Bovine Serum
FGF	Fibroblast Growth Factor
IL	Interleukin
MMP	Matrix Metalloproteinase
*n*	number of experimental replicates
NF-kb	Nuclear Factor kappa B
NCI	Matrix Metalloproteinase
PEMn	Precipitated Extracellular Marennine
RTCA	Real Cell Time Analysis
SDF-1	Stromal Cell-Derived Factor 1
VEGF -R	Vascular Endothelial Growth Factor Receptor
